# Neutral Sodium Humate Modulates Growth, Slaughter Traits, Antioxidant Status, and Gut Health in Yellow-Feathered Broilers

**DOI:** 10.3390/ani15142142

**Published:** 2025-07-19

**Authors:** Junran Lv, Mingzhu Cen, Benkuan Li, Xin Feng, Hongyu Cai, Huihua Zhang

**Affiliations:** College of Animal Science and Technology, Foshan University, Foshan 528225, China; 15303088773@163.com (J.L.); cenmingzhu@126.com (M.C.); lbk9583@163.com (B.L.); 3haofx@gmail.com (X.F.); 13229226214@163.com (H.C.)

**Keywords:** yellow-feathered broilers, growth performance, intestinal morphology, gut microbiota, antioxidant capacity

## Abstract

In this study, we explored the dose-dependent effects of neutral sodium humate (NSH) on the growth performance, slaughter traits, antioxidant capacity, and intestinal health of yellow-feathered broilers. A total of 240 one-day-old male yellow-feathered broilers were randomly allocated into three groups, with each group consisting of four replicates containing 20 birds per replicate. The control group (Blank) received a basal diet, while the NSH-L and NSH-H groups were provided with the same basal diet included with neutral sodium humate at concentrations of 0.15% and 0.3%, respectively. The results indicate that dietary inclusion of neutral sodium humate at both low (NSH-L) and high (NSH-H) doses significantly increased the body weight and leg muscle yield of yellow-feathered broilers and improved meat quality (*p* < 0.05). Furthermore, NSH inclusion effectively reduced serum total cholesterol levels and elevated total protein concentration *(p* < 0.05). In addition, NSH significantly improved duodenal and ileal morphology (e.g., increased villus height and reduced crypt depth), strengthened intestinal barrier integrity *(p* < 0.05), and enhanced antioxidant capacity (*p* < 0.05). Notably, the high-dose NSH group (NSH-H) demonstrated significantly greater enhancements in intestinal and barrier integrity compared to the low-dose group (NSH-L). Microbiome analysis revealed that, compared to the Blank group, both the NSH-L and NSH-H groups exhibited significant shifts in cecal microbiota composition, including increased abundance of *Bacteroidota* and *Rikenellaceae_RC9_gut_group* and reduced abundance of *Euryarchaeota* and *Methanobrevibacter*. Collectively, these findings demonstrate that neutral sodium humate acts as a multifunctional feed additive in yellow-feathered broilers, enhancing productivity and gut health. The study provides a theoretical foundation for the scientific application of neutral sodium humate in broiler production.

## Simple Summary

Sodium humate is a natural humic substance with antioxidant properties and intestinal health-promoting effects, which may enhance poultry health and productivity. In this study, neutral sodium humate (NSH), derived from sodium humate through pH adjustment to create a more neutral formulation, was used to include in the diets of yellow-feathered broilers at concentrations of 0.15% and 0.3%. The results demonstrated that neutral sodium humate inclusion improved intestinal morphology, enhanced gut barrier function, modulated gut microbiota balance, and boosted antioxidant capacity, thereby promoting growth performance. Additionally, meat quality parameters were significantly improved. These findings suggest that neutral sodium humate can serve as a functional feed additive to support broiler health and production efficiency.

## 1. Introduction

The livestock industry has long consumed large quantities of grain as animal feed in the process of meat production [1]. However, this pattern of increased food consumption not only raises production costs but also places potential strain on the food supply chain. To achieve cost reduction and improved efficiency, researchers and farming enterprises have been exploring various strategies, with the use of feed additives, including both biological and chemical agents, gaining widespread attention [2]. By incorporating these additives, feed utilization can be significantly enhanced, leading to reduced feed consumption and improved economic efficiency in livestock production [3,4,5].

Sodium humate is a multifunctional polymer compound, typically extracted from lignite, weathered coal, and peat. It is composed of humic acid (HA), combustion residues, water, and water-soluble substances [6,7]. As a natural organic compound derived from humus, sodium humate has gained increasing attention as a feed additive due to its broad biological activity and low toxicity [4,8]. Sodium humate has been shown to improve poultry egg quality and boost immune function [9,10]. It also improves the antioxidant capacity in weaned piglets [11]. Additionally, sodium humate may help mitigate the negative effects of heavy metal pollution on poultry [12], and it has been found to significantly improve both growth performance and slaughter performance [13,14]. However, while there has been considerable research into its effects on various aspects of poultry health, studies specifically focusing on the impact of neutral sodium humate (NSH) remain limited.

NSH is derived from sodium humate through pH adjustment, resulting in a more neutral formulation. This property distinguishes NSH from standard sodium humate, which tends to be more alkaline. The near-neutral pH of NSH may make it a milder and potentially more compatible additive for animal gastrointestinal systems. Although no studies have yet investigated the use of NSH as a feed additive in poultry, it is hypothesized that its gentler pH profile may exert less irritation on the intestinal tract and possibly contribute to improved gut health. However, due to the current lack of direct evidence, its specific biological effects and mechanisms of action remain to be elucidated. Therefore, this study aims to explore the potential impacts of NSH inclusion on broiler chickens, with a particular focus on growth performance, serum biochemical parameters, antioxidant capacity, intestinal health, and meat quality.

## 2. Materials and Methods

### 2.1. Ethics Statement

The experimental protocol was approved by the Animal Care and Use Committee of Foshan University (Approval No.: FOSU#19-025).

### 2.2. Experimental Design and Housing

The experiment was conducted at the Foshan University Poultry Research Farm, utilizing a two-tier overlapping cage system equipped with vertical ventilation exhaust fans. The experimental chickens were provided by Guangzhou Muyuan Poultry Industry Co., Ltd. (Guangzhou, China). A total of 240 healthy, disease-free, fast-growing, yellow-feathered male broilers, 1 day old, were randomly assigned to three groups: the control group (Blank), which was fed a corn-soybean meal-based basal diet, and two experimental groups—the low-dose NSH group (NSH-L) and the high-dose NSH group (NSH-H)—which were fed diets including 0.15% and 0.3% of NSH, respectively, from a 60% purity NSH product. Each treatment group had 4 replicates, with 20 birds per replicate. The trial lasted for 57 days and was divided into three phases: Starter (1–21 days), Grower (22–42 days), and Finisher (43–57 days). The basal diet was scientifically formulated according to the Chinese Feed Composition Table (30th edition) and the “Nutrient Requirements of Yellow-Feathered Broilers” standard (NY/T 3645-2020) [15]. The composition and nutrient levels of the basal diet are presented in Table 1. The 0.15% and 0.3% NSH (60% content) used in the experiment were provided by Slon Biotechnology Co., Ltd., Beijing, China.

During the experiment, birds had free access to water and feed, with continuous lighting (24 h a day). For the first three days, the room temperature was maintained between 33 °C and 34 °C, and then it was gradually reduced by 3 °C each week until it reached 24 °C at the end of the third week. The humidity was consistently controlled between 60% and 70% throughout the experiment.

### 2.3. Growth Performance

Broilers were weighed at 8:00 AM on days 1, 22, 43, and 57, with measurements taken according to the replicates. Prior to weighing, the chickens were fasted for 12 h, but water was not restricted. Daily feed intake was recorded, and the average daily feed intake, average daily weight gain, and feed conversion ratio (FCR) were calculated. Mortality was recorded throughout the experimental period.

### 2.4. Serum Biochemical and Antioxidant Indices

On days 22, 43, and 57 of the trial, the chickens were fasted for 12 h with free access to water. Two birds from each replicate cage, with weights close to the average, were selected for blood collection. Approximately 5 mL of blood was drawn via venipuncture, then centrifuged at 3000× *g* and 4 °C for 10 min. The serum was carefully extracted, aliquoted, and stored at −20 °C for later analysis. The concentrations of total cholesterol (TCH), alkaline phosphatase (AKP), total protein (TP), and albumin (ALB) were measured [16]. In addition, antioxidant indicators, including total superoxide dismutase (T-SOD), glutathione peroxidase (GSH-Px), malondialdehyde (MDA), and total antioxidant capacity (T-AOC), were also determined. All measurements were performed using commercial kits provided by the Nanjing Institute of Bioengineering (Nanjing Jiancheng Bioengineering Institute, Nanjing, Jiangsu, China) [16].

### 2.5. Intestinal Tissue Morphology

The chickens were humanely euthanized after blood collection by severing the connection between the head and the cervical vertebrae to ensure rapid death. Samples of the duodenum, jejunum, and ileum were collected by trained personnel and immediately fixed in 4% formalin solution. The samples were then dehydrated, cleared, and embedded in paraffin. Serial sections of 5 μm thickness were prepared, and two consecutive sections from each paraffin block were deparaffinized with xylene, rehydrated, and stained with hematoxylin and eosin (H and E). The stained sections were observed under a light microscope equipped with a 10 × objective lens and connected to a high-resolution digital imaging system (LEICA DFC290, Leica Microsystems, Heerbrugg, Switzerland). Measurements were taken from relatively intact and vertically oriented villi along with their corresponding crypt depths [17].

### 2.6. cDNA Synthesis from Extracted RNA

On day 57, mucosal samples from the ileum and jejunum of euthanized and bled chickens were collected using a sterile scalpel and immediately placed into RNase-free tubes. The samples were then flash-frozen in liquid nitrogen and stored at −80 °C. Total RNA was extracted from each sample using TransZol reagent (TransGen Biotech, Beijing, China). RNA concentration was measured using a VWRI732-2534 spectrophotometer (Avantor, Radnor, PA, USA). A 1 μL aliquot of the extracted RNA solution was used to determine RNA concentration, as well as the OD260/280 and OD260/230 ratios. If the OD260/280 ratio was between 1.8 and 2.0, the RNA sample was considered intact and suitable for further experiments. For cDNA synthesis, 1 μg of RNA was treated with the RT EasyTM II (with gDNase) Kit (Foregene, Chengdu, China) to remove genomic DNA contamination, followed by reverse transcription to synthesize cDNA, which was then stored at −20 °C [17].

### 2.7. Quantitative PCR

The primers for specific genes were designed using the NCBI Primer Tool (http://www.ncbi.nlm.nih.gov, accessed on 1 December 2024), and these primers were confirmed to be specific. The primer sequences for the genes are listed in Table 2. The primers were synthesized by Qingke Biotechnology Co., Ltd. (Beijing, China). Gene expression analysis was performed on a real-time quantitative PCR system (QuantStudio 3, Thermo Fisher Scientific, Shanghai, China) using the Real-Time PCR EasyTM-SYBR Green I Kit (Foregene, Chengdu, China). The barrier-related genes tested included *mucin-2* (*MUC-2*), *mucin-1* (*MUC-1*), *ZO-1*, *claudin-1*, and *occludin*, with *β-actin* used as the reference gene and the control group as the reference sample. The relative gene expression levels were calculated using the 2-ΔΔCt method [17].

### 2.8. Slaughter Performance

At 57 days of age, one bird with an average body weight was selected from each replicate (four birds per treatment group) for slaughter performance analysis. Prior to slaughter, birds were fasted for 12 h to empty their digestive tracts, after which live body weight was recorded. The birds were then humanely euthanized, scalded, and defeathered. The head, viscera, and feet were removed in accordance with standard procedures.

Slaughter yield refers to the weight of the bird after the removal of feathers, blood, and the keratinized layers of the feet and beak.

Semi-eviscerated yield is measured after the removal of internal organs including the trachea, esophagus, crop, intestines, spleen, pancreas, reproductive organs, gallbladder, gizzard contents, and the gizzard cuticle membrane.

Fully eviscerated yield is obtained after complete removal of all internal organs, including the heart, liver, proventriculus (glandular stomach), cleaned gizzard, and abdominal fat, as well as the head and feet.

Abdominal fat yield is determined by collecting and weighing all visible fat deposits surrounding the gizzard, proventriculus, cloaca, and within the abdominal cavity.

Gizzard yield is measured after the gizzard contents and cuticle membrane have been thoroughly removed.

Breast muscle yield is calculated by dissecting and weighing the large pectoral muscles located on both sides of the bird’s sternum.

Leg muscle yield is obtained by dissecting and weighing the thigh and drumstick muscles from both legs. Slaughter performance indicators were determined according to the Chinese standard NY/T 823-2004 [22], Terminology and Statistical Methods for Poultry Production Performance [23].

### 2.9. Meat Quality

To evaluate meat quality, several parameters were measured. The pH of breast muscle was determined at 45 min, 24 h, and 48 h postmortem using a pH probe (Testo 205, AG, Lenzkirch, Germany), with samples stored immediately after slaughter at 0–4 °C for 48 h. Each measurement was repeated three times and averaged. After pH measurement, meat color was assessed using a Minolta reflectance colorimeter (CR-300, Konica Minolta Sensing, Osaka, Japan), also in triplicate. The water loss rate was measured by cutting breast muscle samples along the muscle fibers into 3.0 cm × 3.0 cm pieces, weighing them (W1), and then suspending them in containers at 4 °C for 24 and 48 h before reweighing (W2). Drip loss was calculated using the formula: water loss rate (%) = 100 × (W1 − W2)/W1, with three replicates averaged for each sample. Finally, shear force was assessed by cutting the breast muscle into 2.0 cm × 2.0 cm sections and measuring using a digital tenderness meter (C-LM3, Bulader Co., Beijing, China), with three repeated measurements averaged for each sample [24].

### 2.10. Intestinal Microbial Analysis

On day 57, cecal microbiota analysis was performed on euthanized chickens. After extracting the cecal contents, they were briefly frozen with liquid nitrogen and then rapidly cooled to −80 °C. DNA was extracted, and the 16S rRNA gene was amplified using Phusion™ High-Fidelity PCR Master Mix. The PCR products were analyzed by 2% agarose gel electrophoresis, and qualified products were purified using magnetic beads. Quantification was performed using enzyme-linked assays, and the samples were pooled in equal amounts for further electrophoresis and target band recovery. After constructing the library, its quality was assessed using Qubit and Q-PCR. Once confirmed, paired-end sequencing (PE250) was carried out on the NovaSeq6000 platform (lllumina, San Diego, CA, USA). Data were split according to the barcode and primer sequences. After merging the paired-end data, primers were removed using Cutadapt, and quality control was conducted with fastp software (Version 0.23.1), resulting in clean tag data for subsequent analysis [25]. By aligning the sequences with the SILVA and Unite databases, chimeric sequences were removed, resulting in high-quality effective tags (Effective Tags) for downstream analysis [26]. Finally, the data were denoised using the DADA2 module of QIIME2 (Version QIIME2-202202), resulting in the identification of amplicon sequence variants (ASVs) and the construction of a feature table [27], Species annotation analysis was performed using the Silva138.1 database. In QIIME2, alpha diversity analysis (including Shannon, Simpson, Chao1, Good’ s coverage, Dominance, and Pielou indices) and beta diversity analysis were conducted. PCoA analysis was performed using NovoMagic for calculation and visualization.

### 2.11. Data Analysis

Statistical analysis was performed using IBM SPSS Statistics (version 20, IBM Corp., Armonk, NY, USA) software. The normality of the distribution of data in each treatment was assessed using the Shapiro–Wilk test, and the homogeneity of variance between treatments was tested using Levene’s test. A one-way analysis of variance (ANOVA) followed by Bonferroni-corrected post-hoc multiple comparison tests was conducted to determine significant differences between groups. Results are presented as mean ± standard error of the mean (x ± SEM), with *p* > 0.05 indicating no statistically significant difference and *p* < 0.05 indicating a significant difference. The 16S rDNA sequencing data were analyzed using the Novomagic sequencing platform (https://magic.novogene.com/, accessed on 1 November 2024), and some results were visualized using GraphPad Prism 8 software.

## 3. Results

### 3.1. Growth Performance

Growth performance metrics are displayed in Table 3. On day 22, no significant differences were observed between the groups for any of the parameters. On day 43, the final body weight (FBW) of the NSH-H group was significantly higher than that of the control group (*p* < 0.05). On day 57, both the NSH-L and NSH-H groups showed significantly higher FBW compared to the control group (*p* < 0.05). Throughout the entire experimental period (days 1–57), both the NSH-L and NSH-H groups exhibited significantly higher FBW and average daily gain (ADG) compared to the control group (*p* < 0.05). However, there were no significant differences in FCR between the groups (*p* > 0.05).

### 3.2. Antioxidant Indices and Serum Biochemical Indices

The antioxidant indices are shown in Table 4. On day 22, GSH-Px in the NSH-L and NSH-H groups, T-SOD concentration in the NSH-H group, and T-AOC in the NSH-L group were significantly elevated. Additionally, the MDA concentration in the NSH-L group was significantly lower (*p* < 0.05). On day 43, the GSH-Px concentration in both the NSH-L and NSH-H groups was significantly higher, and the MDA concentration showed significant improvement. The T-AOC concentration was significantly increased only in the NSH-H group (*p* < 0.05). On day 57, the T-SOD concentration was significantly higher in both the NSH-L and NSH-H groups, and the MDA concentration showed significant improvement. The GSH-Px concentration was significantly higher only in the NSH-L group.

Serum Biochemical Indices are displayed in Table 5. On day 22, the TP concentration was significantly higher in the NSH-L and NSH-H groups (*p* < 0.05), while no significant differences were observed in the other groups. On day 43, the TP concentration was significantly higher in the NSH-L group (*p* < 0.05), with no significant differences in the other groups. On day 57, the TCH concentration was significantly lower, and the TP concentration was significantly higher in both the NSH-L and NSH-H groups (*p* < 0.05), while no significant differences were observed in the other groups.

### 3.3. Intestinal Morphology and Intestinal Mucosal Barrier Function

Intestinal morphology is displayed in Table 6. In the duodenum, the villus length was significantly increased in both the NSH-L and NSH-H groups (*p* < 0.01), while no significant differences were observed in the other parameters. In the jejunum, no significant differences were found in any of the intestinal parameters between the groups (*p* > 0.05). In the ileum, the villus length in the NSH-H group was significantly higher than in the other groups (*p* < 0.001), with no significant differences observed in the other parameters (*p* > 0.05).

**Figure 1:**
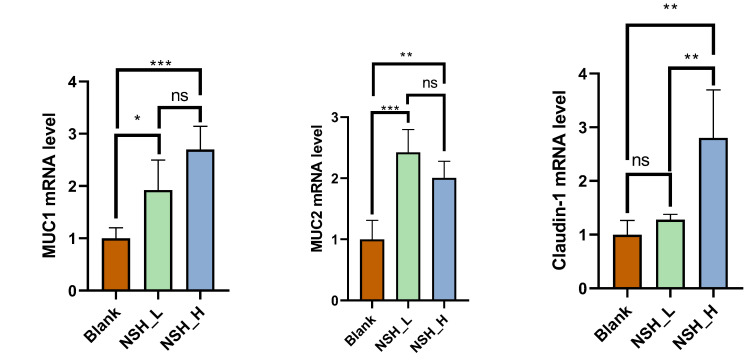

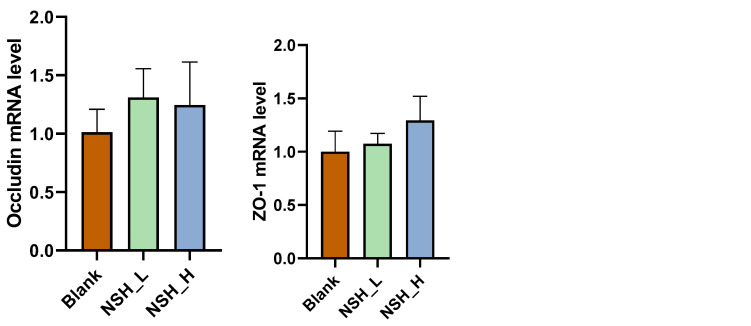
Effects of adding 0.15% and 0.3% NSH to the diet on the mRNA expression of *MUC-1*, *MUC-2*, *occludin*, *claudin-1*, and *ZO-1* in the jejunum. Note: values are expressed as a mean and SEM, with *n* = 4. Asterisks describe differences between groups, where *** indicates a difference significant at *p* < 0.001, ** at *p* < 0.01, and * at *p* < 0.05, and ns indicates no significance.

The intestinal mucosal barrier function of the jejunum is shown in Figure 1. In the jejunum, the mRNA expression of *MUC1* and *MUC2* was significantly increased in both the NSH-L and NSH-H groups (*p* < 0.05). Additionally, the mRNA expression of *Claudin-1* was significantly higher in the NSH-H group compared to the other groups (*p* < 0.01). No significant differences were observed in the expression of the remaining genes.

**Figure 2:**
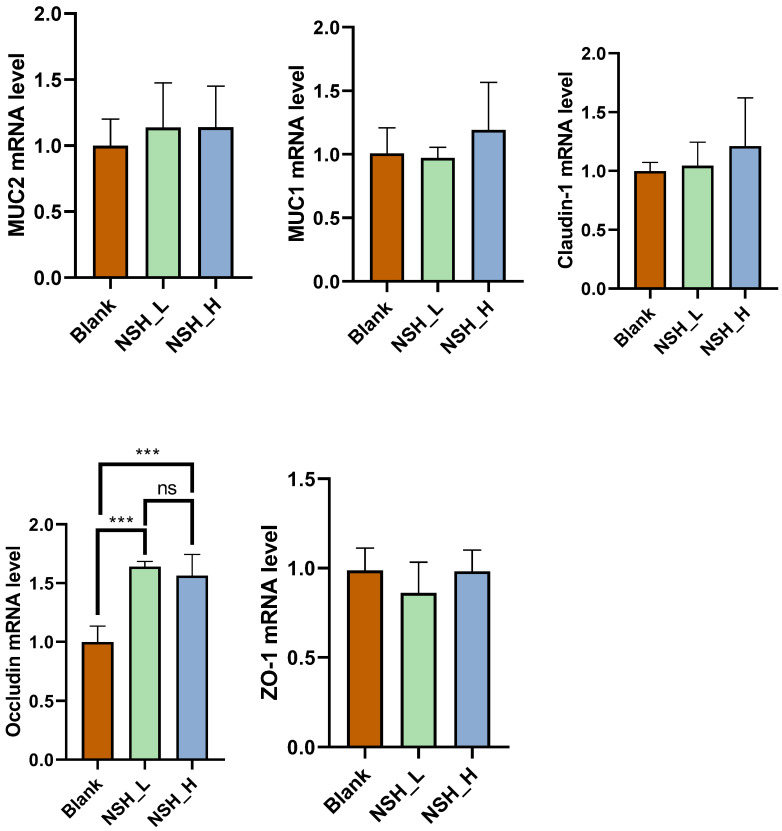
Effects of adding 0.15% and 0.3% NSH to the diet on the mRNA expression of *MUC-1*, *MUC-2*, *occludin*, *claudin-1*, and *ZO-1* in the ileum. Note: Values are expressed as a mean and SEM, with *n* = 4. Asterisks indicate differences between groups, where *** denotes a difference significant at *p* < 0.001, and ns indicates no significance.

The intestinal mucosal barrier function of the ileum is shown in Figure 2. In the ileum, the mRNA expression of *occludin* was significantly higher in both the NSH-L and NSH-H groups (*p* < 0.001), while no significant differences were found in the expression of the other genes.

### 3.4. Carcass Traits and Meat Quality

Carcass traits are displayed in Table 7. Compared to the control group, the leg muscle percentage was significantly higher in both the NSH-L and NSH-H groups (*p* < 0.05). However, no significant differences were observed in the carcass weight percentage, semi-eviscerated carcass percentage, whole eviscerated carcass percentage, gizzard weight percentage, or breast muscle weight percentage (*p* > 0.05).

Meat quality is displayed in Table 8. The 48-h drip loss and pH values were significantly improved in both the NSH-L and NSH-H groups. Additionally, the b* value of meat color (at 45 min, 24 h, and 48 h) was significantly reduced (*p* < 0.05). Moreover, the pH value of the NSH-H group at 24 h was also significantly increased. No significant differences were observed in the other parameters.

### 3.5. Analysis of Intestinal Microorganisms

#### OTU Diversity, Beta Diversity, and Alpha Diversity Analysis

**Figure 3:**
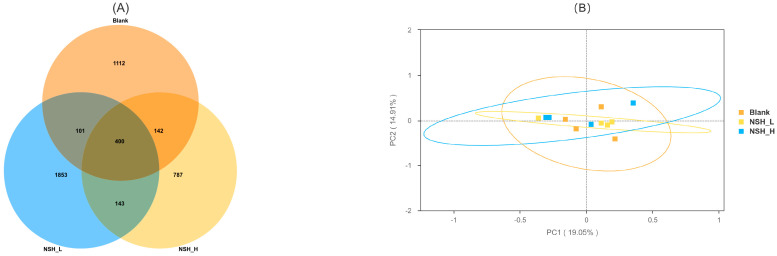
Microbial composition of cecal contents. (**A**) Venn diagram showing the number of operational taxonomic units (OTUs). (**B**) Principal coordinate analysis (PCoA) of the cecal microbiota.

Figure 3 displays (A) a Venn diagram showing the number of operational taxonomic units (OTUs) and (B) a principal coordinate analysis (PCoA) of the cecal microbiota. In this study, a total of 4528 operational taxonomic units (OTUs) were identified, with 400 OTUs shared across all groups. Specifically, the Blank group (A), NSH-L group (J), and NSH-H group (K) contained 1106, 1851, and 780 unique OTUs, respectively. Furthermore, the number of shared OTUs between the Blank group (A) and the NSH-L group (J), as well as between the Blank group (A) and the NSH-H group (K), was 102 and 147, respectively. The shared OTUs between the NSH-L group (J) and the NSH-H group (K) were 142. To further analyze the differences between the samples, principal coordinate analysis (PCoA) was performed using the Bray-Curtis similarity method. The results showed that the first and second principal components explained 19.05% and 14.9% of the variation between the samples, respectively.

**Figure 4:**
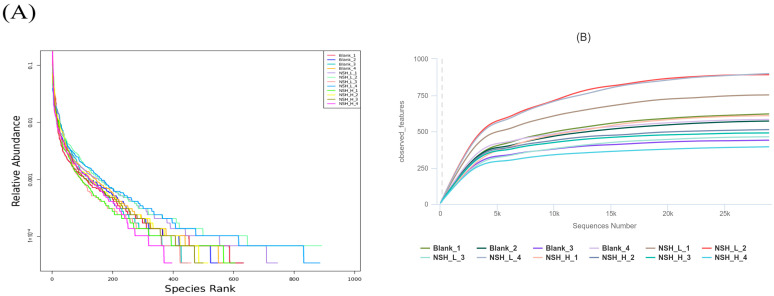
Relationship between microbial community species richness, sequence number, and feature count. (**A**) Relative distribution of species abundance. (**B**) Changes in the number of observed features with increasing sequencing depth.

The rank-abundance curve reflects both species abundance and species evenness. Species abundance is represented by the length of the curve along the horizontal axis; the greater the range along the axis, the higher the species abundance. Species evenness is represented by the shape (smoothness) of the curve; the flatter the curve, the higher the species evenness. As shown in Figure 4A, the horizontal fluctuations of the curve are relatively broad and eventually level off, indicating that the sample composition is both uniform and rich. As shown in Figure 4B, with an increase in sequencing depth, the number of OTUs gradually increases and eventually plateaus, suggesting that the data volume is appropriate.

**Figure 5:**
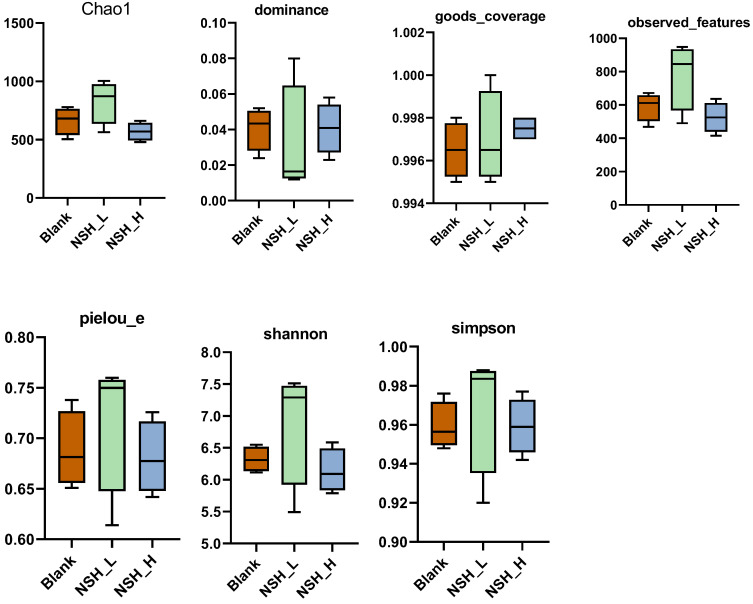
Chao1, observed features, dominance, Good’s coverage, Pielou’s evenness (e), Shannon, and Simpson indices.

Figure 5 displays the alpha diversity analysis. Alpha diversity analysis was conducted to assess the microbial community diversity. There were no significant differences (*p* > 0.05) between groups for the indices of Chao1, observed features, dominance, Good’s coverage, Pielou’s evenness (e), Shannon, and Simpson.

### 3.6. Taxonomic Composition of Cecal Microbiota

**Figure 6:**
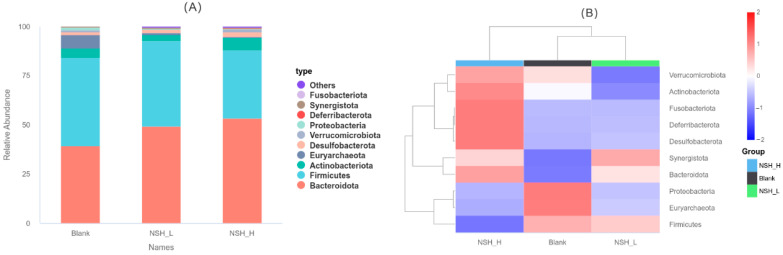
Relative abundance of gut microbiota at the phylum level. (**A**) Bar chart of species abundance at the phylum level. (**B**) Cluster heatmap of phylum-level abundance.

As shown in (A) and (B) of Figure 6, at the phylum level, *Bacteroidota*, *Firmicutes*, *Actinobacteriota*, *Euryarchaeota*, *Desulfobacterota*, *Verrucomicrobiota*, *Proteobacteria*, *Deferribacterota*, *Synergistota*, and *Fusobacteriota* were the top 10 dominant phyla in the cecum across all groups. Compared to the Blank group, the abundance of *Bacteroidota* was significantly higher in both the NSH-L and NSH-H groups, while the abundance of *Euryarchaeota* and *Proteobacteria* was significantly lower.

**Figure 7:**
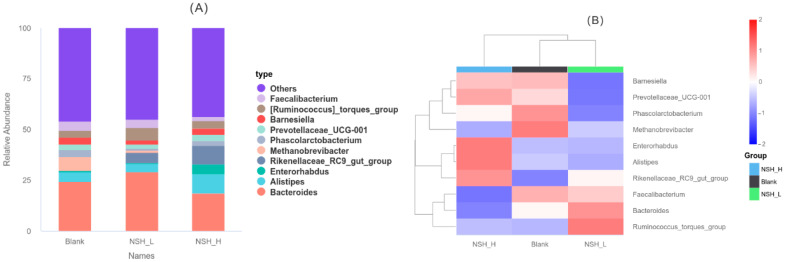
Relative abundance of gut microbiota at the genus level. (**A**) Bar chart of species abundance at the genus level. (**B**) Cluster heatmap of genus-level abundance.

As shown in (A) and (B) of Figure 7, at the genus level, Bacteroides, *Alistipes*, *Enterorhabdus*, *Rikenellaceae_RC9_gut_group*, *Methanobrevibacter*, *Phascolarctobacterium*, *Prevotellaceae_UCG-001*, *Barnesiella*, *[Ruminococcus]HRNHRN_torques_group*, and *Faecalibacterium* were the top 10 dominant genera in the cecum across all groups. Compared to the Blank group, the abundance of *Rikenellaceae_RC9_gut_group* was significantly higher in both the NSH-L and NSH-H groups, while the abundance of *Methanobrevibacter* was significantly lower.

## 4. Discussion

Sodium humate, as a functional feed additive, has attracted increasing attention in recent research [14]. Studies have shown that sodium humate significantly increases body weight and improves FCR of broilers between 22 and 42 days of age [28]. Some studies have also shown that adding 5 g or 10 g of humate per kilogram of feed can have a negative impact on the production performance of broilers [29]. This study found that both FBW and ADG were significantly higher (*p* < 0.05) in the NSH-L group (1.5 g/kg) and the NSH-H group (3 g/kg). These findings suggest that appropriate levels of sodium humate can enhance nutrient absorption, while excessive addition may lead to nutrient imbalances or metabolic burdens.

Commercial poultry production is influenced by various stressors, which often have negative effects on production performance, reproductive performance, and overall health [30,31]. Recent studies have shown that excessive production of free radicals, impaired antioxidant defense mechanisms, and oxidative stress are typically the main causes of the negative consequences of stress in poultry [32]. HA exhibits strong antioxidant activity by inducing the activity of total antioxidant enzymes, catalase, and glutathione reductase, protecting cells from oxidative damage and stress, while also reducing MDA levels [33]. Similarly, the addition of 2 g/kg of sodium humate to the diet was found to significantly increase T-SOD and T-AOC in the serum of piglets while also reducing MDA content [11]. This study also found that NSH significantly increased the activity of GSH-Px, T-AOC, and T-SOD in the blood of yellow-feathered broilers, while simultaneously reducing the MDA concentration in their blood. These effects are likely due to active functional groups in HA, such as carboxyl, hydroxyl, carbonyl, and quinone groups [34], which may also help maintain the integrity of intestinal tissues by mitigating oxidative stress.

The liver plays a critical role in cholesterol regulation; when cholesterol conversion to bile acids is impaired, serum cholesterol rises and may further damage hepatic tissues [35]. In this study, NSH inclusion significantly reduced serum total cholesterol without affecting total protein levels, implying a potential hepatoprotective role. These findings suggest that NSH may support liver function by regulating lipid metabolism, which complements its beneficial effects on nutrient absorption and growth.

The surface of the small intestine is covered with villi and microvilli, structures that significantly increase its surface area. The epithelial cells on the villi are equipped with numerous microvilli, further expanding the contact area and thereby enhancing the efficiency of nutrient absorption [36,37]. Studies have found that when sodium humate is used in combination with probiotics, it can improve growth performance, carcass characteristics, and the morphological features of the intestine [38]. In this study, we observed that sodium humate inclusion significantly increased the villus height in both the duodenum (NSH-L and NSH-H groups) and ileum (NSH-H group), compared to the control group. Tight junction proteins are composed of multiple protein complexes, including occludins, claudins, and zonula occludens proteins, which play a crucial role in maintaining the integrity and function of the epithelial barrier. Previous studies have shown that damage to the epithelial barrier can negatively impact feed efficiency and animal weight gain [39]. Research shows that sodium humate alleviates LPS-induced jejunal mucosal damage by regulating intestinal immune function and optimizing the balance of gut microbiota, helping to maintain the integrity of the intestinal barrier [40]. This experiment also found that the mRNA expression of mucins *MUC1* and *MUC2* in the jejunum was significantly increased in both the NSH-L and NSH-H groups. Additionally, the mRNA expression of the tight junction protein *Claudin-1* was significantly higher in the NSH-H group compared to the other groups (*p* < 0.05). In the ileum, the mRNA expression of *occludin* was also significantly elevated in both the NSH-L and NSH-H groups (*p* < 0.05), while no significant differences were observed in the expression of other genes. NSH improves intestinal morphology and barrier function, enhancing nutrient absorption and reducing energy loss associated with inflammation, thereby promoting growth and health in broilers. The high-dose group (NSH-H) showed more significant improvements in intestinal integrity and barrier function, indicating that higher doses provide greater benefits for overall health and performance.

Moreover, enhanced nutrient uptake and metabolic efficiency were associated with improved carcass traits. Specifically, the leg muscle rate of yellow-feathered broilers was significantly increased in the NSH inclusion groups (*p* < 0.05), aligning with previous findings that NSH in drinking water may positively affect carcass composition [13]. This suggests that NSH-mediated improvements in gut and liver function may contribute to enhanced muscle development.

Today, broiler production has become highly industrialized, and the current focus is on improving various traits of broilers to enhance meat quality [41]. pH is one of the key parameters for assessing meat quality. Lower pH values can lead to a reduction in water-holding capacity of the meat and increased muscle juice loss (drip loss) during storage [42,43]. Generally speaking, higher drip loss may reduce the water-holding capacity and tenderness of the meat [44,45]. In both the NSH-L and NSH-H groups, it was observed that when the pH value of the breast meat after 48 h in the experimental groups was significantly higher than that in the Blank group, the drip loss of the breast meat after 48 h in the NSH-L and NSH-H groups was also significantly lower than that in the control group (*p* < 0.05). This suggests that NSH may improve the water-holding capacity of the meat and reduce drip loss by slowing down the decrease in pH. This study also found that the b values of breast muscle color at 45 min, 24 h, and 48 h were significantly lower in both the NSH-L and NSH-H groups compared to the control group. The b * value is typically associated with the yellowness of the meat; a reduction in yellowness may be related to changes in fatty acid composition, pigment deposition, or other physiological responses. These effects likely stem from improved antioxidant capacity and systemic metabolic balance.

At the microbial level, the gut microbiota plays a critical role in nutrient metabolism and immune regulation [46]. While this study found no significant differences in α- or β-diversity indices among groups, changes in specific taxa were observed. Within the gastrointestinal microbiota, Bacteroidetes possess exceptionally broad metabolic capabilities and are considered one of the most stable components of the gut microbial community [47]. Bacteria within the phylum *Bacteroidota* play a key role in the digestion of complex carbohydrates, fiber, and other organic matter in the gut [48,49,50,51]. They also produce beneficial short-chain fatty acids, such as acetate, propionate, and butyrate [52,53,54], which are essential for maintaining intestinal health, immune regulation, and metabolic stability in the host. An increasing number of studies suggest that *Proteobacteria* may be one of the microbial features associated with disease [55]. *Proteobacteria* are often found in increased numbers in various diseases, and some researchers suggest that their increase may be a potential marker of microbiota instability, which could predispose individuals to a range of diseases [56]. At the genus level, *Rikenellaceae_RC9_gut_group* has been shown to be closely associated with the degradation of structural carbohydrates [57]. Structural carbohydrates are the primary chemical components of most plant tissues and plant cells, aside from water [58]. Therefore, *Rikenellaceae_RC9_gut_group* may play a crucial role in the gut, particularly in the degradation of structural carbohydrates, helping the host digest and utilize the indigestible components in plants. The genus *Methanobrevibacter* is typically spherical or rod-shaped, existing individually, but most commonly appears in pairs or short chains [59]. Among them, *Methanobrevibacter smithii* is considered the dominant methane-producing archaea in the human gastrointestinal tract [59]. However, research on the *Methanobrevibacter* genus remains limited, and its specific role in health and disease is still not well understood. Therefore, the increase in the abundance of the *Bacteroidota* phylum and the *Rikenellaceae_RC9_gut_group* genus, along with the decrease in the abundance of Proteobacteria, may help enhance the metabolic capacity of the gut, improve nutrient absorption, and promote the overall health of the host. Together, these microbial shifts may synergize with the improved intestinal morphology and antioxidant capacity, further supporting the enhanced growth performance observed in NSH-inclusion broilers.

## 5. Conclusions

In summary, inclusion of NSH at both low (NSH-L) and high (NSH-H) doses may promote the growth performance of yellow-feathered broilers by optimizing the intestinal morphology of the duodenum and ileum, enhancing the barrier function of the jejunum and ileum, and improving antioxidant capacity. Moreover, the high-dose group exhibited more pronounced effects in improving intestinal morphology and barrier function. The experimental groups (NSH-L and NSH-H) also significantly increased the leg muscle percentage and total protein levels, reduced total cholesterol in the serum, and may have improved muscle hydration by maintaining muscle pH, thereby reducing drip loss and enhancing meat quality. In terms of gut microbiota, compared to the Blank group, both the NSH-L and NSH-H groups significantly increased the abundance of *Bacteroidota* and *Rikenellaceae_RC9_gut_group*, while reducing the abundance of *Euryarchaeota* and *Methanobrevibacter*. This suggests that NSH can optimize gut health and metabolic function by modulating the structure of the gut microbiota, increasing the abundance of beneficial bacteria (such as *Bacteroidota* and *Rikenellaceae_RC9_gut_group*), and reducing the abundance of methane-producing archaea (such as *Methanobrevibacter*).

## Figures and Tables

**Table 1:** Feed composition offered to broilers during the experiment.

Ingredients (100%)	1–21 d	22–42 d	43–56 d
Corn	44.50	39.50	42.20
Soybean meal (CP = 46%)	33.50	33.00	27.70
Extruded soybean	5.00	0.00	0.00
Sorghum	10.00	20.00	20.00
Lard	1.50	3.50	6.40
Fish meal	1.30	0.00	0.00
Dicalcium phosphate	1.30	1.30	1.20
Limestone	1.40	1.40	1.00
L-lysine hydrochloride	0.25	0.19	0.24
DL-methionine	0.23	0.21	0.19
L-threonine	0.05	0.06	0.05
^1^ Premix compound	0.71	0.57	0.75
Sodium chloride	0.12	0.15	0.15
Choline chloride	0.12	0.10	0.10
^2^ Antioxidant	0.01	0.01	0.01
Phytase	0.01	0.01	0.01
Calculated nutrient composition			\
Crude protein	21.45	19.09	17.11
Metabolizable Energy (kcal/kg)	2877.00	2956.05	3173.48
Crude fat	4.87	6.02	8.85
Calcium	1.00	0.94	0.75
Total Phosphorus	0.64	0.60	0.56
Lysine	1.39	1.16	1.06
Methionine	0.56	0.49	0.45
Threonine	0.88	0.78	0.69

^1^ Premix provided per kilogram of the diet: vitamin A 12,000 IU; vitamin D_3_ 3000 IU; vitamin E 10 IU; vitamin K_3_ 2 mg; vitamin B_1_ 1 mg; vitamin B_2_ 3 mg; vitamin B_6_ 2 mg; vitamin B_12_ 0.01 mg; niacin 20 mg; pantothenic acid 4 mg; folic acid 0.54 mg; biotin 0.05 mg; Fe 100 mg; Cu 20 mg; Mn 100 mg; Zn 80 mg; I 3 mg; Se 0.5 mg. ^2^ Antioxidant: The antioxidants used in the premix are Butylated Hydroxytoluene (BHT) and Tert-butylhydroquinone (TBHQ).

**Table 2:** Primer sequences used in quantitative real-time PCR.

Genes	Primer Sequence	Temperature (℃)	Number	Reference
*β-actin*	F: CATTGTCCACCGCAAATGCT R: AAGCCATGCCAATCTCGTCT	57.2	NM_205518.1	[18]
*Occludin*	F: CGGAGCCCAGACTACCAAAG R: TTACACAGCTTCAGCCTTACA	55.8	XM_025144247.1	[18]
*Claudin-1*	F: GGTATGGCAACAGAGTGGCT R: CAGCCAATGAAGAGGGCTGA	57.0	NM_001013611.2	[18]
*MUC-2*	F: TTCATGATGCCTGCTCTTGTG R: CCTGAGCCTTGGTACATTCTTGT	58.0	XM_421035	[19]
*ZO-1*	F: CTTCAGGTGTTTCTCTTCCTCCTC R: CTGTGGTTTCATGGCTGGATC	56.6	XM_413773	[20]
*ZO-2*	F: AGTGGCCACCATTGTTGTGA R:ACTGTAGCCACTTCGAGCAC	56.2	XM_046934790	[21]

**Table 3:** The growth performance of broilers.

Items	Treatment			SEM	*p*-Value
	Blank	NSH-L	NSH-L		
Initial Body Weight (g)	32.26 ± 0.05	32.26 ± 0.06	32.23 ± 0.05	0.015	0.548
22 d FBW (g)	372.96 ± 4.85	372.35 ± 9.48	384.39 ± 5.17	2.443	0.059
1–22 d ADG (g/d)	16.22 ± 0.23	16.19 ± 0.45	16.76 ± 0.24	0.116	0.062
1–22 d Average Daily Feed Intake (g/d)	27.53 ± 0.94	26.3 ± 0.94	28.06 ± 1.59	0.383	0.159
1–22 d FCR	1.7 ± 0.05	1.61 ± 0.04	1.68 ± 0.08	0.019	0.122
43 d FBW (g)	1272.75 ± 14.02 ^a^	1302.14 ± 15.31 ^ab^	1310.64 ± 18.38 ^b^	6.436	0.021
23–43 d ADG (g/d)	42.84 ± 0.77	44.27 ± 1.14	44.1 ± 0.65	0.298	0.091
23–43 d Average Daily Feed Intake (g/d)	71.28 ± 1.89	71.76 ± 0.99	72.13 ± 0.53	0.348	0.649
23–43 d FCR	1.64 ± 0.05	1.62 ± 0.02	1.63 ± 0.02	0.009	0.679
57 d FBW (g)	1949.77 ± 13.29 ^a^	1995.96 ± 10.64 ^b^	2006.07 ± 6.31 ^b^	7.879	<0.001
44–57 d ADG (g/d)	48.35 ± 0.99	49.55 ± 1.07	49.67 ± 1.06	0.326	0.197
44–57 d Average Daily Feed Intake (g/d)	133.38 ± 3.01	130.61 ± 1.19	131.97 ± 1.42	0.632	0.214
44–57 d FCR	2.71 ± 0.06	2.64 ± 0.08	2.72 ± 0.17	0.031	0.607
1–57 d ADG (g/d)	34.24 ± 0.24 ^a^	35.07 ± 0.19 ^b^	35.25 ± 0.11 ^b^	0.141	<0.001
1–57 d Average Daily Feed Intake (g/d)	70.4 ± 1.18	69.43 ± 0.45	70.56 ± 0.48	0.254	0.14
1–57 d FCR	2.03 ± 0.04	1.98 ± 0.02	2.01 ± 0.02	0.01	0.113

Note: Values are expressed as a mean and SEM, with *n* = 4. Significant differences between groups are indicated by different lowercase letters (^a,b^) in superscripts (*p* < 0.05), while the same or no letters denote no significant differences (*p* > 0.05).

**Table 4:** The antioxidant indicators of broiler.

Items	Treatment			SEM	*p*-Value
	Blank	NSH-L	NSH-H		
22 days					
GSH-Px (U/mgprot)	2009.51 ± 119.56 ^a^	2697.11 ± 184.1 ^c^	2387.31 ± 123.46 ^b^	92.89	<0.001
T-SOD (U/mL)	366.31 ± 6.68 ^a^	379.21 ± 15.16 ^a^	427.7 ± 34.06 ^b^	9.8	0.008
T-AOC (mM)	0.23 ± 0.02 ^a^	0.37 ± 0.06 ^b^	0.29 ± 0.03 ^ab^	0.02	0.003
MDA (nmol/mL)	4.92 ± 0.24 ^a^	3.06 ± 0.49 ^b^	4.31 ± 0.63 ^a^	0.264	0.001
43 days					
GSH-Px (U/mgprot)	392.88 ± 13.41 ^a^	590.79 ± 29.28 ^b^	561.8 ± 11.84 ^b^	26.82	<0.001
T-SOD (U/mL)	171.09 ± 8.81	182.17 ± 7.73	168.81 ± 14.13	3.28	0.217
T-AOC (mM)	0.44 ± 0.02 ^a^	0.48 ± 0.05 ^a^	0.78 ± 0.08 ^b^	0.048	<0.001
MDA (nmol/mL)	5.06 ± 0.46 ^a^	2.81 ± 0.33 ^b^	2.78 ± 0.26 ^b^	0.335	<0.001
57 days					
GSH-Px (U/mgprot)	852.14 ± 16.65 ^a^	921.65 ± 4.31 ^b^	868.72 ± 6.52 ^a^	9.36	<0.001
T-SOD (U/mL)	129.35 ± 3.48 ^a^	167.39 ± 7.45 ^b^	167.41 ± 6.28 ^b^	5.63	<0.001
T-AOC (mM)	0.39 ± 0.02	0.45 ± 0.04	0.46 ± 0.02	0.015	0.102
MDA (nmol/mL)	3.94 ± 0.33 ^a^	2.83 ± 0.5 ^b^	2.93 ± 0.33 ^b^	0.183	0.006

Abbreviations: GSH-Px (U/mgprot): glutathione peroxidase, T-SOD (U/mL): total superoxide dismutase, T-AOC (mM): total antioxidant capacity, MDA (nmol/mL): malondialdehyde. Note: values are expressed as a mean and SEM, with *n* = 4. Significant differences between groups are indicated by different lowercase letters (^a,b,c^) in superscripts (*p* < 0.05), while the same or no letters denote no significant differences (*p* > 0.05).

**Table 5:** The serum biochemical parameters of broiler.

Items	Treatment			SEM	*p*-Value
	Blank	NSH-L	NSH-H		
22 days					
TCH (mmol/L)	5.83 ± 0.99	5.83 ± 0.39	5.92 ± 0.7	0.193	0.982
ALB (g/L)	17.27 ± 0.42	18.36 ± 1.97	19.67 ± 1.66	0.493	0.133
TP (g/L)	28.73 ± 0.76 ^a^	32.85 ± 2.02 ^b^	32.39 ± 1.84 ^b^	0.7	0.012
AKP (Kinyoun Units/100 mL)	90.81 ± 31.64	66.74 ± 7.71	60.51 ± 6.34	6.36	0.114
43 days					
TCH (mmol/L)	4.66 ± 1.06	3.59 ± 0.79	4.38 ± 0.85	0.273	0.273
ALB (g/L)	13.01 ± 0.72	12.05 ± 0.45	12.53 ± 1.47	0.282	0.425
TP (g/L)	19.49 ± 0.58 ^a^	24.9 ± 2.49 ^b^	22.03 ± 1.51 ^ab^	0.802	0.005
AKP (Kinyoun Units/100 mL)	69.61 ± 18.31	65.23 ± 21.43	65.67 ± 17.77	5.06	0.94
57 days					
TCH (mmol/L)	5.78 ± 0.31 ^a^	3.82 ± 0.64 ^b^	4.56 ± 0.35 ^b^	0.272	0.001
ALB (g/L)	14.55 ± 0.98	14.06 ± 0.61	15.2 ± 0.25	0.227	0.113
TP (g/L)	24.1 ± 0.34 ^a^	27.01 ± 0.86 ^b^	27.72 ± 0.84 ^b^	0.509	<0.001
AKP (Kinyoun Units/100 mL)	64.34 ± 14.05	56.91 ± 18.24	48.31 ± 12.29	4.4	0.364

Abbreviations: TCH (mmol/L): total cholesterol, ALB (g/L): albumin, TP (g/L): total protein, AKP (Kinyoun Units/100 mL): Alkaline Note: values are expressed as a mean and SEM, with *n* = 4. Significant differences between groups are indicated by different lowercase letters (^a,b^) in superscripts (*p* < 0.05), while the same or no letters denote no significant differences (*p* > 0.05).

**Table 6:** The intestinal tissue morphology of broilers.

Items	Treatment			SEM	*p*-Value
	Blank	NSH-L	NSH-H		
Duodenum					
VH (μm)	1686.55 ± 25.26 ^a^	1851.91 ± 73.3 ^b^	1862.72 ± 49.11 ^b^	27.97	0.002
CD (μm)	98.75 ± 3.73	104.57 ± 14.89	114.29 ± 10.6	3.412	0.176
V:C	17.09 ± 0.48	18 ± 2.77	16.42 ± 1.82	0.541	0.537
Jejunum					
VH (μm)	1455.66 ± 20.83	1562.04 ±89.94	1533.73 ± 53.41	21.04	0.089
CD (μm)	121.83 ± 36.48	94.96 ± 14.34	98.54 ± 6.95	6.994	0.252
V:C	12.98 ± 4.73	16.8 ± 3.18	15.61 ± 0.99	0.996	0.303
Ileum					
VH (μm)	1221.21 ± 48.52 ^a^	1170.64 ± 76.13 ^a^	1422.35 ± 44.93 ^b^	36.13	<0.001
CD (μm)	111.77 ± 8.00	97.82 ± 17.88	99.1 ± 13.58	4.063	0.33
V:C	10.98 ± 1.11	12.29 ± 2.42	14.57 ± 2.08	0.678	0.077

Note: values are expressed as a mean and SEM, with *n* = 4. Significant differences between groups are indicated by different lowercase letters (^a,b^) in superscripts (*p* < 0.05), while the same or no letters denote no significant differences (*p* > 0.05).

**Table 7:** The carcass traits of broilers.

Items	Treatment			SEM	*p*-Value
	Blank	NSH-L	NSH-H		
Slaughter Yield %	91.3 ± 0.85	92.03 ± 3.33	89.55 ± 0.76	0.617	0.258
Semi-eviscerated Yield %	80.27 ± 1.11	80.56 ± 3.43	78.38 ± 2.86	0.752	0.482
Fully Eviscerated Yield%	65.33 ± 2.21	65.46 ± 2.2	64.24 ± 1.57	0.552	0.656
Abdominal Fat Yield%	2.63 ± 0.07	2.39 ± 0.25	2.33 ± 0.22	0.065	0.125
Breast Muscle Yield%	8.84 ± 0.88	9.26 ± 0.86	9.58 ± 0.38	0.108	0.41
Leg Muscle Yield%	12.5 ± 0.92 ^a^	14.16 ± 0.68 ^b^	14.12 ± 0.74 ^b^	0.156	0.024

Note: Values are expressed as a mean and SEM, with *n* = 4. Significant differences between groups are indicated by different lowercase letters (^a,b^) in superscripts (*p* < 0.05), while the same or no letters denote no significant differences (*p* > 0.05).

**Table 8:** The meat quality of broilers.

Items	Treatment			SEM	*p*-Value
	Blank	NSH-L	NSH-H		
Shear force (N)	4.39 ± 0.73	4.78 ± 0.25	4.4 ± 0.44	0.145	0.503
Drip loss 24 h	2.46 ± 0.45	1.83 ± 0.51	1.81 ± 0.1	0.138	0.079
Drip loss 48 h	6.17 ± 0.57 ^a^	4.92 ± 0.15 ^b^	4.61 ± 0.46 ^b^	0.232	0.001
PH45min	6.22 ± 0.21	6.48 ± 0.31	6.26 ± 0.17	0.071	0.297
PH24h	5.66 ± 0.03 ^a^	5.71 ± 0.05 ^ab^	5.79 ± 0.08 ^b^	0.022	0.036
PH48h	5.59 ± 0.02 ^a^	5.68 ± 0.03 ^b^	5.73 ± 0.02 ^b^	0.182	<0.001
L45min	44.38 ± 1.11	47.29 ± 2.32	47.14 ± 2.1	0.644	0.105
a45min	1.89 ± 0.34	1.64 ± 0.9	1.23 ± 0.29	0.172	0.318
b45min	12.32 ± 0.98 ^a^	9.94 ± 0.78 ^b^	9.53 ± 0.66 ^b^	0.427	0.002
L24h	52.76 ± 1.21	53.34 ± 1.58	51.85 ± 1.09	0.389	0.316
a24h	1.46 ± 0.29	1.36 ± 0.28	1.16 ± 0.21	0.078	0.33
b24h	14.15 ± 0.63 ^a^	12.3 ± 0.71 ^b^	11.95 ± 0.49 ^b^	0.332	0.001
L48h	52.95 ± 3.14	54.71 ± 1.41	52.68 ± 3.23	0.762	0.545
a48h	1.22 ± 0.28	1.07 ± 0.31	1.06 ± 0.1	0.069	0.625
b48h	14.67 ± 0.39 ^a^	12.14 ± 1.34 ^b^	12.12 ± 1.17 ^b^	0.454	0.011

Note: values are expressed as a mean and SEM, with *n* = 4. Significant differences between groups are indicated by different lowercase letters (^a,b^) in superscripts (*p* < 0.05), while the same or no letters denote no significant differences (*p* > 0.05).

## Data Availability

The original contributions presented in this study are included in the article. Further inquiries can be directed to the corresponding author.

## References

[1] Wirsenius S., Azar C., Berndes G. (2010). How much land is needed for global food production under scenarios of dietary changes and livestock productivity increases in 2030?. Agric. Syst..

[2] Wang J., Deng L., Chen M., Che Y., Li L., Zhu L., Chen G., Feng T. (2024). Phytogenic feed additives as natural antibiotic alternatives in animal health and production: A review of the literature of the last decade. Anim. Nutr..

[3] Ali A., Ponnampalam E.N., Pushpakumara G., Cottrell J.J., Suleria H.A., Dunshea F.R. (2021). Cinnamon: A natural feed additive for poultry health and production—A review. Animals.

[4] Arif M., Alagawany M., Abd El-Hack M., Saeed M., Arain M., Elnesr S. (2019). Humic acid as a feed additive in poultry diets: A review. Iran. J. Vet. Res..

[5] Abd El-Hack M.E., Alagawany M., Arif M., Emam M., Saeed M., Arain M.A., Siyal F.A., Patra A., Elnesr S.S., Khan R.U. (2018). The uses of microbial phytase as a feed additive in poultry nutrition—A review. Ann. Anim. Sci..

[6] Ji Y., Zhang A., Chen X., Che X., Zhou K., Wang Z. (2016). Sodium humate accelerates cutaneous wound healing by activating TGF-β/Smads signaling pathway in rats. Acta Pharm. Sin. B.

[7] Wang D., You Z., Du Y., Zheng D., Jia H., Liu Y. (2022). Influence of sodium humate on the growth performance, diarrhea incidence, blood parameters, and fecal microflora of pre-weaned dairy calves. Animals.

[8] Jooné G.K., Dekker J., van Rensburg C.E.J. (2003). Investigation of the immunostimulatory properties of oxihumate. Z. Naturforsch. C.

[9] El-Ratel I.T., El Basuini M.F., Khattab A.A., Mekawy A.I., Fouda S.F. (2023). Ameliorative impacts of sodium humate on heat-stressed laying Japanese quail (*Coturnix coturnix* Japonica). Anim. Physiol. Anim. Nutr..

[10] Zhang A., Pirzado S., Liu G., Chen Z., Chang W., Cai H., Bryden W., Zheng A. (2020). Dietary supplementation with sodium humate improves egg quality and immune function of laying hens. J. Appl. Anim. Nutr..

[11] Wang Q., Ying J., Zou P., Zhou Y., Wang B., Yu D., Li W., Zhan X. (2020). Effects of dietary supplementation of humic acid sodium and zinc oxide on growth performance, immune status and antioxidant capacity of weaned piglets. Animals.

[12] Skalická M., Koréneková B., Nad P., Makóová Z. (2002). The role of natrium humate on cadmium elimination and copper level in poultry. Chem. Inż. Ekol..

[13] Proskina L., Barzdina D., Valdovska A., Pilvere I., Vircava I., Cerina S., Meskis S. (2023). Assessment of the inclusion of a feed additive of sodium humate derived from freshwater sapropel in diets for broiler chickens. Vet. World.

[14] Li C., Li X., Li P., Wei B., Zhang C., Zhu X., Jie Z. (2022). Sodium humate alters the intestinal microbiome, short-chain fatty acids, eggshell ultrastructure, and egg performance of old laying hens. Front. Vet. Sci..

[15] (2020). Yellow-Feathered Broiler Chicken Nutritional Requirements.

[16] Hou J., Lu L., Lian L., Tian Y., Zeng T., Ma Y., Li S., Chen L., Xu W., Gu T. (2024). Effects of coated sodium butyrate on the growth performance, serum biochemistry, antioxidant capacity, intestinal morphology, and intestinal microbiota of broiler chickens. Front. Microbiol..

[17] Su Y., Wang M., Wu Z., Huang P., Zeng J. (2025). Dihydrosanguinarine enhances tryptophan metabolism and intestinal immune function via AhR pathway activation in broilers. J. Anim. Sci. Biotechnol..

[18] Wang Q., Zhan X., Wang B., Wang F., Zhou Y., Xu S., Li X., Tang L., Jin Q., Li W. (2022). Modified montmorillonite improved growth performance of broilers by modulating intestinal microbiota and enhancing intestinal barriers, anti-inflammatory response, and antioxidative capacity. Antioxidants.

[19] Wang H., Liang S., Li X., Yang X., Long F., Yang X. (2019). Effects of encapsulated essential oils and organic acids on laying performance, egg quality, intestinal morphology, barrier function, and microflora count of hens during the early laying period. Poult. Sci..

[20] Liu Y., Zhang S., Luo Z., Liu D. (2021). Supplemental Bacillus subtilis PB6 improves growth performance and gut health in broilers challenged with Clostridium perfringens. J. Immunol. Res..

[21] Zheng C., Xiao G., Yan X., Qiu T., Liu S., Ou J., Cen M., Gong L., Shi J., Zhang H. (2023). Complex of lauric acid monoglyceride and cinnamaldehyde ameliorated subclinical necrotic enteritis in yellow-feathered broilers by regulating gut morphology, barrier, inflammation and serum biochemistry. Animals.

[22] (2004). Nomenclature of Poultry Production Performance Terms and Statistical Methods.

[23] Chen K., Lv J., Luo Z., Liu Z., Cen M., Li B., Ou J., Zhang H. (2025). The effect of amylase, chromium propionate and their combination supplementation on growth performance, carcass traits, serum parameters, antioxidant capacity and intestinal health in yellow feathered broilers. Poult. Sci..

[24] Wang Z., Xing T., Zhang L., Zhao L., Gao F. (2024). Effects of substituting soybean meal with fermented rapeseed meal mixture on the growth performance, slaughter performance, meat quality, blood biochemical indices and intestinal barrier function in Langshan Chickens. Poult. Sci..

[25] Bokulich N.A., Subramanian S., Faith J.J., Gevers D., Gordon J.I., Knight R., Mills D.A., Caporaso J.G. (2013). Quality-filtering vastly improves diversity estimates from Illumina amplicon sequencing. Nat. Methods.

[26] Edgar R.C., Haas B.J., Clemente J.C., Quince C., Knight R. (2011). UCHIME improves sensitivity and speed of chimera detection. Bioinformatics.

[27] Wang Y., Guo H., Gao X., Wang J. (2021). The intratumor microbiota signatures associate with subtype, tumor stage, and survival status of esophageal carcinoma. Front. Oncol..

[28] Kocabağli N., Alp M., Acar N., Kahraman R. (2002). The effects of dietary humate supplementation on broiler growth and carcass yield. Poult. Sci..

[29] Hassan S. (2014). Effect of adding dietary humate on productive performance of broiler chicks. CABI Digit. Libr..

[30] Surai P., Fisinin V. (2016). Vitagenes in poultry production: Part 1. Technological and environmental stresses. World’s Poult. Sci. J..

[31] Surai P., Fisinin V. (2016). Vitagenes in poultry production: Part 2. Nutritional and internal stresses. World’s Poult. Sci. J..

[32] Surai P.F., Kochish I.I., Fisinin V.I., Kidd M.T. (2019). Antioxidant Defence Systems and Oxidative Stress in Poultry Biology: An Update. Antioxidants.

[33] Kamel M.M., Elhady M., El Iraqi K., Wahba F. (2015). Biological immune stimulants effects on immune response, behavioural and productive performance of broilers. Egypt. Poult. Sci..

[34] de Melo B.A.G., Motta F.L., Santana M.H.A. (2016). Humic acids: Structural properties and multiple functionalities for novel technological developments. Mater. Sci. Eng. C.

[35] Tabas I. (2002). Cholesterol in health and disease. J. Clin. Investig..

[36] Schneeman B.O. (2002). Gastrointestinal physiology and functions. Br. J. Nutr..

[37] Kiela P.R., Ghishan F.K. (2016). Physiology of intestinal absorption and secretion. Best Pract. Res. Clin. Gastroenterol..

[38] Eivollahi L., Ahady M.T., Sahraei M. (2019). The Effect of Sodium Humate and Probiotic on Performance, Carcass traits, Immunological Indices and Gut Morphology in Broiler Chickens. Vet. Res..

[39] Kogut M.H., Genovese K.J., Swaggerty C.L., He H., Broom L. (2018). Inflammatory phenotypes in the intestine of poultry: Not all inflammation is created equal. Poult. Sci..

[40] He Y., Wang D., Liu K., Deng S., Liu Y. (2023). Sodium humate alleviates LPS-induced intestinal barrier injury by improving intestinal immune function and regulating gut microbiota. Mol. Immunol..

[41] Mir N.A., Rafiq A., Kumar F., Singh V., Shukla V. (2017). Determinants of broiler chicken meat quality and factors affecting them: A review. J. Food Sci. Technol..

[42] Chmiel M., Słowiñski M., Janakowski S. (2014). The quality evaluation of RFN and PSE pork longissimus lumborum muscle considering its microstructure. Ann. Anim. Sci..

[43] Jankowiak H., Cebulska A., Bocian M. (2021). The relationship between acidification (pH) and meat quality traits of polish white breed pigs. Eur. Food Res. Technol..

[44] Pearce K.L., Rosenvold K., Andersen H.J., Hopkins D.L. (2011). Water distribution and mobility in meat during the conversion of muscle to meat and ageing and the impacts on fresh meat quality attributes—A review. Meat Sci..

[45] Disetlhe A.R., Marume U., Mlambo V., Hugo A. (2018). Effects of dietary humic acid and enzymes on meat quality and fatty acid profiles of broiler chickens fed canola-based diets. Asian-Australas. J. Anim. Sci..

[46] Yadav S., Jha R. (2019). Strategies to modulate the intestinal microbiota and their effects on nutrient utilization, performance, and health of poultry. J. Anim. Sci. Biotechnol..

[47] Shetty S.A., Hugenholtz F., Lahti L., Smidt H., de Vos W.M. (2017). Intestinal microbiome landscaping: Insight in community assemblage and implications for microbial modulation strategies. FEMS Microbiol. Rev..

[48] Wexler A., Goodman A. (2017). An insider’s perspective: Bacteroides as a window into the microbiome. Nat. Microbiol..

[49] Dehority B. (1969). Pectin-fermenting bacteria isolated from the bovine rumen. J. Bacteriol..

[50] Bryant M. (1974). Nutritional features and ecology of predominant anaerobic bacteria of the intestinal tract. Am. J. Clin. Nutr..

[51] Salyers A., Vercellotti J., West S., Wilkins T. (1977). Fermentation of mucin and plant polysaccharides by strains of Bacteroides from the human colon. Appl. Environ. Microbiol..

[52] Horvath T.D., Ihekweazu F.D., Haidacher S.J., Ruan W., Engevik K.A., Fultz R., Hoch K.M., Luna R.A., Oezguen N., Spinler J.K. (2022). *Bacteroides ovatus* colonization influences the abundance of intestinal short chain fatty acids and neurotransmitters. iScience.

[53] Rios-Covian D., Arboleya S., Hernandez-Barranco A.M., Alvarez-Buylla J.R., Ruas-Madiedo P., Gueimonde M., de los Reyes-Gavilan C.G. (2013). Interactions between Bifidobacterium and Bacteroides species in cofermentations are affected by carbon sources, including exopolysaccharides produced by bifidobacteria. Appl. Environ. Microbiol..

[54] Rios-Covian D., Sánchez B., Salazar N., Martínez N., Redruello B., Gueimonde M., de Los Reyes-Gavilán C.G. (2015). Different metabolic features of Bacteroides fragilis growing in the presence of glucose and exopolysaccharides of bifidobacteria. Front. Microbiol..

[55] Rizzatti G., Lopetuso L., Gibiino G., Binda C., Gasbarrini A. (2017). Proteobacteria: A common factor in human diseases. BioMed Res. Int..

[56] Shin N.-R., Whon T.W., Bae J.-W. (2015). Proteobacteria: Microbial signature of dysbiosis in gut microbiota. Trends Biotechnol..

[57] Pitta D.W., Pinchak W.E., Dowd S.E., Osterstock J., Gontcharova V., Youn E., Dorton K., Yoon I., Min B.R., Fulford J.D. (2010). Rumen bacterial diversity dynamics associated with changing from bermudagrass hay to grazed winter wheat diets. Microb. Ecol..

[58] Reid J.G., Dey P.M., Harborne J.B. (1997). Carbohydrate metabolism: Structural carbohydrates. Plant Biochemistry.

[59] Mohammadzadeh R., Mahnert A., Duller S., Moissl-Eichinger C. (2022). Archaeal key-residents within the human microbiome: Characteristics, interactions and involvement in health and disease. Curr. Opin. Microbiol..

